# The Prevalence of Depressive and Insomnia Symptoms, and Their Association With Quality of Life Among Older Adults in Rural Areas in China

**DOI:** 10.3389/fpsyt.2021.727939

**Published:** 2021-09-22

**Authors:** Juan-Juan Yang, Hong Cai, Lei Xia, Weicheng Nie, Yulong Zhang, Song Wang, Yudong Shi, Chee H. Ng, Huanzhong Liu, Yu-Tao Xiang

**Affiliations:** ^1^Department of Psychiatry, School of Mental Health and Psychological Sciences, Anhui Medical University, Hefei, China; ^2^Department of Psychiatry, Chaohu Hospital of Anhui Medical University, Hefei, China; ^3^Unit of Psychiatry, Department of Public Health and Medicinal Administration, Institute of Translational Medicine, Faculty of Health Sciences, University of Macau, Taipa, Macao, SAR China; ^4^Centre for Cognitive and Brain Sciences, University of Macau, Taipa, Macao, SAR China; ^5^Institute of Advanced Studies in Humanities and Social Sciences, University of Macau, Taipa, Macao, SAR China; ^6^Department of Psychiatry, The Melbourne Clinic and St Vincent's Hospital, University of Melbourne, Richmond, VA, Australia

**Keywords:** depression, insomnia, older adults, quality of life, rural areas

## Abstract

**Background:** There are few studies on the epidemiology of depression, insomnia, and their association with quality of life (QOL) in older adults living in rural China. This study examined the prevalence of depressive and insomnia symptoms, and their association with QOL in community-dwelling older adults in a rural area in Anhui province, China.

**Methods:** This was a cross-sectional study conducted in the rural areas of four cities (Hefei, Huaibei, Anqing, and Xuancheng) in Anhui province between July and October, 2019 using random sampling method. All community-dwelling residents from the selected villages who met the study entry criteria were invited to participate in this study. Depressive and insomnia symptoms and QOL were assessed with the Chinese version of self-reported Center for Epidemiological Survey Depression Scale (CES-D), the Insomnia Severity Index (ISI) and the 26-item World Health Organization Quality of Life Brief version (WHOQOL-BREF), respectively.

**Results:** A total of 871 older adults were included. The prevalence of overall depressive symptoms, insomnia symptoms, and comorbid depressive and insomnia symptoms were 34.0% [95% confidence intervals (95% CI): 30.8–37.1%], 45.7% (95% CI: 42.4–49.0%) and 20.3% (95% CI: 17.6–23.0%), respectively. Older adults with depressive symptoms, insomnia symptoms, and comorbid depressive and insomnia symptoms had lower scores in QOL compared to those without. Depressive symptoms were positively associated with living with families [Odd Ratio (OR) = 1.82, 95% CI: 1.31–2.54] and negatively associated with current drinking (OR = 0.49, 95% CI: 0.33–0.72). Insomnia symptoms were negatively associated with fair and good financial status (fair: OR = 0.53, 95% CI = 0.38–0.75; good: OR = 0.30, 95% CI = 0.14–0.64) and current drinking (OR = 0.64, 95% CI = 0.45–0.93), and positively associated with more frequent major medical conditions (OR = 1.32, 95% CI = 1.16–1.51). Comorbid depressive and insomnia symptoms were positively associated with living with families (OR = 2.02, 95% CI = 1.36–3.00), and negatively associated with fair and good financial status (fair: OR = 0.61, 95% CI = 0.41–0.89; good: OR = 0.34, 95% CI = 0.12–0.95) and current drinking (OR = 0.57, 95% CI = 0.35–0.92).

**Conclusion:** Depressive and insomnia symptoms were common in older adults living in rural areas in China. Considering the negative health outcomes caused by depressive and insomnia symptoms, regular screening and effective treatments should be developed for this population.

## Introduction

Aging population is an increasing trend in many parts of the world. It was estimated that about 80% of older people globally will be living in low- and middle-income countries by 2050 (1). As the largest developing country, China has around 179 million population older than 65 years as of April 2021 (2), and the number will reach up to 390 million by 2050 (3). Compared to their younger counterparts, older adults are more likely to suffer from both physical and mental health problems (4).

Depressive symptoms (depression hereafter) are the most common mental health problem in older adults (4), which is associated with a range of negative health consequences such as an increased risk of suicide (5), frailty (6) and impaired cognition function (7). The prevalence of depression in older adults ranged greatly between different countries and even between areas within a country. For instance, the prevalence of depression ranged from 6.35% in Wuhan (8) to 60.3% in Fuzhou (9), with a higher rate observed in rural areas of China (10). A meta-analysis found that the prevalence of depression in older adults showed an ascending tendency (11). In order to reduce the negative impact of depression on daily functioning and quality of life (QOL) (12), it is necessary to understand the epidemiology of depression in older adults, particularly in rural areas.

Insomnia symptoms (insomnia hereafter) are common health problems in older adults, and are often associated with physical diseases, psychiatric disorders and related problems such as poor attention and memory, depression and anxiety (13), falls (14), and growing health costs (15). The overall prevalence of insomnia in older adults between studies varied from 30 to 62.1% (16–18). Older adults living in rural areas are more likely to suffer from insomnia than their urban counterparts (19).

Compared to their younger counterparts, older adults are more likely to suffer from physical diseases and psychiatric problems. Many older adults have poor social and family support because of separation from their children, particularly in rural areas of China (20). Furthermore, due to limited mental health awareness (21) and mental health services in rural areas (22), psychiatric problems in older adults, such as depressive and insomnia symptoms, are often not identified. In addition, as a widely used outcome measure that reflects self-perception toward overall health status of life, QOL and its association with depressive and insomnia symptoms have been inadequately studied in rural older adults in China.

Anhui province, located in the central area of China, is one of the major agricultural provinces nationwide, with its rural population accounting for 44.2% of the whole population (23). With such high proportion of rural older adults, Anhui is representative of rural areas of China in terms of family structure and access to health services (24). In recent decades, due to the rapid migration many young people in Anhui province have moved to large cities in eastern China, their parents/grandparents often find themselves living alone, i.e., the so-called “empty nests” syndrome (25) which may result in mental health problems. Therefore, to reduce the negative health outcomes caused by depressive and insomnia symptoms and allocate health appropriate resources, it is important to understand the prevalence of depressive and insomnia symptoms in this vulnerable population. Previous studies found that insomnia in older adults was positively associated with living alone and having less social contacts and social capital (26), while depression was associated with poverty, female gender, older age, illiteracy, unemployment, presence of chronic physical diseases, and more frequent hospitalizations in older adults in Anhui, China (27). To date, however, the prevalence of comorbid depressive and insomnia symptoms, and their association with QOL have not been reported; additionally, random sampling was not used in most of the previous epidemiological studies (7, 28, 29).

Therefore, we examined the prevalence of depressive and insomnia symptoms, and comorbid depressive and insomnia symptoms, and explored their associations with demographic and clinical characteristics and QOL in older adults living in rural areas of Anhui, China.

## Methods

### Study Sites and Participants

A cross-sectional study was conducted in the rural areas of four cities (Hefei, Huaibei, Anqing, and Xuancheng) in Anhui province in China between July and October 2019. A community-based survey with randomized cluster sampling method was adopted. In each of the four cities, three to five villages were randomly selected using a web-based random number table. All community-dwelling older adults in the selected villages were identified based on the databases in the local community offices (i.e., “*Juweihui*”) using the following inclusion criteria: (1) aged 60 years and above; (2) able to understand the purpose and content of the assessments. Those with obvious cognitive problems (e.g., dementia and severe brain injury) were excluded based on a review of their health records. All participants provided written informed consent. The study protocol was approved by the medical ethics committee of Chaohu Hospital of Anhui Medical University.

### Data Collection and Measurements

A face-to-face interview was conducted in the homes of the participants by trained research assistants. Basic sociodemographic data of the participants, such as gender, age, education, marital status, religion, living status, perceived financial status, current drinking and number of major medical conditions, were collected. Severity of depression symptoms was assessed using the Chinese version of the self-reported Center for Epidemiological Survey Depression Scale (CES-D) (30, 31) with 20 items. The CES-D total score ranges between 0 and 60, with ≥16 as “having depression,” and ≥20 is considered as “having moderate to severe depression” (31). The Chinese version of the self-report Insomnia Severity Index (ISI) (32, 33) was used to assess severity of insomnia. The ISI consists of seven questions scoring from “0 (none)” to “4 (very severe),” with a higher total score indicating more severe insomnia symptoms. The ISI total score of ≥8 is considered as “having insomnia,” ≥15 is considered as “having moderate insomnia,” and ≥22 is considered as “having severe insomnia” (34). The Chinese version of the self-report 26-item World Health Organization Quality of Life Brief version (WHOQOL-BREF) (35, 36) was used to measure QOL in physical, psychological, social and environmental domains. Drinking was defined as drinking at least once per week for the purpose of social communication and activities, with the average total amount of ethanol of over 250 g (equivalent to 250 ml of beer or 50 ml of wine) (37).

### Statistical Analysis

Data analyses were performed using Statistic Package for Social Science (SPSS) version 23.0 (SPSS Inc., Chicago, Illinois, USA). Normal distributions of continuous variables were checked by one-sample Kolmogorov-Smirnoff test. Independent *t*-tests, Mann-Whitney U tests and Chi-square tests were used to compare socio-demographic and clinical variables between depression and no depression groups, between insomnia and no insomnia groups, and between no comorbid depression and insomnia and comorbid depression and insomnia groups, respectively. QOL between depression and no depression groups, between insomnia and no insomnia groups, and between comorbid depression and insomnia and no comorbid depression and insomnia groups were compared using analysis of covariance (ANOVA) after controlling for the potentially confounding effects of variables that significantly differed in univariate analyses. Binary logistic regression analyses with the “Enter” method were used to examine the independent correlates of depression, insomnia, and comorbid depression and insomnia, separately. Variables with significant group differences in univariate analyses were entered as independent variables, while depression, insomnia, and comorbid depression and insomnia were dependent variable, separately. All analyses used the two-tailed tests, with the significance of 0.05.

## Results

### Sample Characteristics

Of 1,029 older adults who were invited to participate in this study, 871 met the study entry criteria and completed the assessment, giving a participation rate of 84.6%. The mean age of the study sample was 70.2 [standardized deviation (SD): 8.9] years, half were males, 27.2% were unmarried, 43.9% had a higher education level (secondary school and above). Their basic demographic characteristics of the study sample are shown in Table 1.

**Table 1:** The sociodemographic and clinical characteristics.

**Variables**	**Total** **(*N* = 871)**	**No DEP** **(*N* = 575)**	**DEP** **(*N* = 296)**	**Univariate analyses**			**No IS** **(*N* = 473)**	**IS** **(*N* = 398)**	**Univariate analyses**			**No comorbid DEP and IS** **(*N* = 694)**	**Comorbid DEP and IS** **(*N* = 177)**	**Univariate analyses**		
	***N*** **(%)**	***N*** **(%)**	***N*** **(%)**	**χ^**2**^**	**df**	***P***	***N*** **(%)**	***N*** **(%)**	**χ^**2**^**	**df**	***P***	***N*** **(%)**	***N*** **(%)**	**χ^**2**^**	**df**	***P***
Male gender	436 (50.1)	314 (54.6)	122 (41.2)	14.0	1	**<0.001**	251 (53.1)	185 (46.5)	3.8	1	0.053	367 (52.9)	69 (39.0)	10.9	1	**0.001**
Unmarried	237 (27.2)	149 (25.9)	88 (29.7)	1.4	1	0.23	112 (23.7)	125 (31.4)	6.5	1	**0.01**	183 (26.4)	54 (30.5)	1.2	1	0.27
Secondary school and above	382 (43.9)	277 (48.2)	105 (35.5)	12.8	1	**<0.001**	235 (49.7)	147 (36.9)	14.3	1	**<0.001**	326 (47.0)	56 (31.6)	13.5	1	**<0.001**
Living with families	504 (57.9)	318 (55.3)	186 (62.8)	4.6	1	**0.03**	275 (58.1)	229 (57.5)	0.03	1	0.86	388 (55.9)	116 (65.5)	5.4	1	**0.02**
Having a religion	164 (18.8)	111 (19.3)	53 (17.9)	0.3	1	0.62	97 (20.5)	67 (16.8)	1.9	1	0.17	134 (19.3)	30 (16.9)	0.5	1	0.47
Perceived financial status	–	–	–	4.5	2	0.11	–	–	14.9	2	**0.001**	–	–	9.9	2	**0.007**
Poor	263 (30.2)	160 (27.8)	103 (34.8)	–	–	–	119 (25.2)	144 (36.2)	–	–	–	193 (27.8)	70 (39.5)	–	–	–
Fair	568 (65.2)	388 (67.5)	180 (60.8)	–	–	–	326 (68.9)	242 (60.8)	–	–	–	466 (67.1)	102 (57.6)	–	–	–
Good	40 (4.6)	27 (4.7)	13 (4.4)	–	–	–	28 (5.9)	12 (3.0)	–	–	–	35 (5.0)	5 (2.8)	–	–	–
Current drinking	236 (27.1)	189 (32.9)	47 (15.9)	28.6	1	**<0.001**	143 (30.2)	93 (23.4)	5.2	1	**0.02**	208 (30.0)	28 (15.8)	14.3	1	**<0.001**
	**M** **(SD)**	**M** **(SD)**	**M** **(SD)**	***t/Z***	**df**	***P***	**M** **(SD)**	**M** **(SD)**	***t/Z***	**df**	***P***	**M** **(SD)**	**M** **(SD)**	***t/Z***	**df**	***P***
Age (years)	70.2 (8.9)	70.2 (8.8)	70.2 (9.2)	−0.001	869	0.999	70.1 (9.0)	70.3 (8.8)	−0.4	869	0.71	70.3 (8.8)	70.1 (9.3)	0.2	869	0.86
Number of major medical conditions	1.2 (1.2)	1.2 (1.2)	1.2 (1.2)	−0.4	^−a^	0.68	1.0 (1.04)	1.4 (1.26)	−4.8	^−a^	**<0.001**	1.2 (1.1)	1.3 (1.2)	−0.8	^−a^	0.40
Physical QOL	13.4 (2.6)	13.9 (2.5)	12.5 (2.5)	8.2	869	**<0.001**	14.2 (2.4)	12.5 (2.6)	10.3	869	**<0.001**	13.8 (2.5)	12.0 (2.7)	8.7	869	**<0.001**
Psychological QOL	13.8 (2.5)	14.4 (2.3)	12.8 (2.4)	9.2	869	**<0.001**	14.3 (2.3)	13.3 (2.5)	6.3	869	**<0.001**	14.1 (2.4)	12.9 (2.5)	5.8	869	**<0.001**
Social QOL	14.6 (2.5)	15.0 (2.4)	13.7 (2.4)	7.8	869	**<0.001**	14.9 (2.3)	14.2 (2.6)	4.4	869	**<0.001**	14.8 (2.4)	13.7 (2.6)	5.5	869	**<0.001**
Environmental QOL	14.1 (2.2)	14.5 (2.1)	13.4 (2.1)	7.1	869	**<0.001**	14.4 (2.0)	13.8 (2.2)	4.3	869	**<0.001**	14.3 (2.1)	13.4 (2.2)	4.7	869	**<0.001**

−a *Mann-Whitney U tests; Bolded value: <0.05*.

*DEP, depression; IS, insomnia; M, mean; SD, standard deviation; QOL, quality of life*.

The prevalence of the overall depression (CES-D total score ≥ 16) was 34.0 [95% confidence intervals (95% CI): 30.8–37.1%], while the prevalence of moderate to severe depression (CES-D total score ≥ 20) was 20.6% (95% CI: 17.9–23.2%). The prevalence of overall insomnia (ISI total score ≥ 8) was 45.7% (95% CI: 42.4–49.0%), while the prevalence of moderate (ISI total score ≥ 15) and severe insomnia (ISI total score ≥ 22) was 15.5% (95% CI: 13.1–17.9%) and 3.7% (95% CI: 2.4–4.9%), respectively. The prevalence of comorbid depression and insomnia was 20.3% (95% CI: 17.6–23.0%).

### Univariate Analyses

As shown in Table 1, patients with depression were less likely to be male (*P* < 0.001), have secondary school and above education (*P* < 0.001), and have current drinking (*P* < 0.001), and more likely to live with families (*p* = 0.03). Patients with insomnia were more likely to be unmarried (*p* = 0.01) and have more frequent major medical conditions (*P* < 0.001), but less likely to have higher education level (secondary school and above) (*P* < 0.001), good financial status (*P* < 0.001) and current drinking (*p* = 0.02). Patients with comorbid depression and insomnia were more likely to live with families (*p* = 0.02), and less likely to be male (*P* = 0.001), and have higher education level (secondary school and above) (*P* < 0.001), good financial stats (*P* = 0.007), and current drinking (*P* < 0.001).

### Multivariate Analyses

ANCOVA revealed that those with depression, insomnia and comorbid depression and insomnia had lower physical [depression group: *F*_(1, 871)_ = 58.5, *P* < 0.001; insomnia group: *F*_(1, 871)_ = 81.4, *P* < 0.001; comorbid depression and insomnia group: *F*_(1, 871)_ = 67.3, *P* < 0.001], psychological [depression group: *F*_(1, 871)_ = 86.0, *P* < 0.001; insomnia group: *F*_(1, 871)_ = 31.5, *P* < 0.001; comorbid depression and insomnia group: *F*_(1, 871)_= 32.8, *P* < 0.001], social [depression group: *F*_(1, 871)_ = 52.5, *P* < 0.001; insomnia group: *F*_(1, 871)_ = 16.6, *P* < 0.001; comorbid depression and insomnia group: *F*_(1, 871)_ = 28.3, *P* < 0.001], and environmental QOL [depression group: *F*_(1, 871)_ = 44.7, *P* < 0.001; insomnia group: *F*_(1, 871)_ = 12.2, *P* < 0.001; comorbid depression and insomnia group: *F*_(1, 871)_ = 18.8, *P* < 0.001] compared to those without.

Table 2 shows the results of binary logistic regression analyses. Depression was positively associated with living with families [Odds Ratio (OR) = 1.82, 95% CI: 1.31–2.54] and negatively associated with current drinking behavior (OR = 0.49, 95% CI: 0.33–0.72). Insomnia was negatively associated with fair and good financial status (fair: OR = 0.53, 95% CI = 0.38–0.75; good: OR = 0.30, 95% CI = 0.14–0.64) and current drinking behavior (OR = 0.64, 95% CI = 0.45–0.93), and positively associated with more frequent major medical conditions (OR = 1.32, 95% CI = 1.16–1.51). Comorbid depression and insomnia were positively associated with living with families (OR = 2.02, 95% CI = 1.36–3.00), and negatively associated with fair and good financial status (fair: OR = 0.61, 95% CI = 0.41–0.89; good: OR = 0.34, 95% CI = 0.12–0.95) and current drinking behavior (OR = 0.57, 95% CI = 0.35–0.92).

**Table 2:** Independent correlates of depression, insomnia, and comorbid depression and insomnia among older adults in Anhui rural areas.

**Variables**	**Depression** ^**a**^			**Insomnia** ^**a**^			**Comorbid depression and insomnia** ^**a**^		
	***P***	**OR**	**95% CI**	***P***	**OR**	**95% CI**	***P***	**OR**	**95% CI**
Male gender	0.13	0.78	0.56–1.08	0.34	0.86	0.62–1.18	0.19	0.77	0.53–1.14
Unmarried	0.05	1.43	1.00–2.05	0.52	1.12	0.79–1.58	0.17	1.33	0.88–2.01
Secondary school and above	0.16	0.79	0.57–1.10	0.43	0.88	0.64–1.21	0.16	0.75	0.51–1.11
Living with families	**<0.001**	1.82	1.31–2.54	0.30	1.18	0.86–1.62	**0.001**	2.02	1.36–3.00
Perceived financial status	–	–	–	–	–	–	–	–	–
Poor	Ref	–	–	Ref	–	–	Ref	–	–
Fair	0.33	0.84	0.60–1.19	**<0.001**	0.53	0.38–0.75	**0.01**	0.61	0.41–0.89
Good	0.42	0.73	0.34–1.56	**0.002**	0.30	0.14–0.64	**0.04**	0.34	0.12–0.95
Current drinking	**<0.001**	0.49	0.33–0.72	**0.02**	0.64	0.45–0.93	**0.02**	0.57	0.35–0.92
Number of major medical conditions	0.47	0.95	0.84–1.09	**<0.001**	1.32	1.16–1.51	0.71	1.03	0.89–1.20

a *Study sites were controlled as covariate; Bolded value: <0.05*.

*CI, confidential interval; OR, odds ratio; Ref, reference group*.

## Discussion

To the best of our knowledge, this was the first study to examine the prevalence of depression, insomnia, and comorbid depression and insomnia, and their association with QOL in older adults living in rural Anhui, China. We found that the prevalence of depression and insomnia were 34.0% and 45.7%, respectively, both of which are consistent with the corresponding figures reported previously in China. For example, earlier studies in rural older adults found that the prevalence of depression as measured by the 30-item Geriatric Depression Scale (GDS-30) was 52.9% (27), and the prevalence of insomnia as measured by the Pittsburgh Sleep Quality Index (PSQI) was 33.8% (38). A meta-analysis (39) revealed that the prevalence of depression in Chinese older adults was 31.0% in rural areas. The high rates of depression in older adults could be partly due to the health and social problems associated with aging process and the fast-changing social structure following the economic development in China such as the “empty nest” syndrome in rural areas. Empty-nest older adults refer to those who have no children or whose children have left home and thus they live alone or with their spouse or older parents (40). Another meta-analysis (19) revealed that the pooled prevalence of insomnia was 44.0% (95% CI: 31.2–56.8%) in Chinese older adults in rural areas, which is similar to our finding. In contrast, the prevalence of depression and insomnia in other countries varied greatly; for example, the corresponding figures of depression varied from 7% in Malaysia (41) and Singapore (42) to 81% in India (43). A meta-analysis of studies in low and middle income countries (44) revealed that the prevalence of insomnia ranged from 9.1% (95% CI: 8–11%) in China to 37.7% (95% CI: 35–40%) in India. Due to different population characteristics such as education level, physical health status, socioeconomic level associated with the epidemiological studies of depression and insomnia (44), direct comparisons between studies should be made with caution.

Compared to the corresponding figures in urban older adults, the prevalence found in our study were generally higher. For instance, previous studies found that the prevalence of depression and insomnia was 10.1% (45) and 30.6% (46), respectively, in Chinese urban older adults. Further, previous meta-analyses found that the prevalence of depression was 22.34% (39), while the prevalence of insomnia was 34.3% (95% CI: 29.1–39.6%) (19) in older people in urban areas of China. The reasons for the lower prevalence of depression and insomnia among older adults in urban areas may include better financial status, greater access to and provision of health services, and better social support (47, 48). However, it should be noted that due to different measurement tools, sampling methods and sampling sources, direct comparisons between studies are problematic.

Comorbid depression and insomnia were common in this study, which is consistent with previous findings (49, 50). A meta-analysis of 23 studies (49) revealed that insomnia could increase the risk of depression in older adults and vice versa. The mechanisms for depression and insomnia (51) are common involving stress (52), dopamine system (53), and inflammatory factors (54). In addition, the decreased neuronal activity of the suprachiasmatic nucleus and disruption of the circadian clock during the aging process may be associated with increased rates of comorbid depression and insomnia in older adults (55, 56).

In this study older adults who lived with families were more likely to have depression and comorbid depression and insomnia, which does not support earlier findings (47, 57) that found living with families appeared to be a protective factor against depression and insomnia in older adults. Several reasons could explain this unexpected finding. First, due to the fast economic development, traditional Chinese family structure has collapsed in the past decades, particularly in rural areas i.e., young people do not live usually together with their parents/grandparents as many of them work in cities resulting in “empty-nest” older adults. However, in the case that older adults suffer from severe physical diseases and/or psychiatric problems, their younger family members usually returned to home to care for the older adults. Second, older adults who lived with their family probably have more frequent physical diseases and poorer mental health than those living alone (58), which may increase the risk of depression and insomnia (47, 59). Finally, family conflicts are more common in those living with family members, which could also increase the likelihood of mental health problems (60).

Compared to those in poor financial status, older adults in fair and good financial status were less likely to have insomnia and comorbid depression and insomnia in this study. This is consistent with the notion that there is a negative association between mental health status and poverty (61). On the one hand, those in good financial state usually have less living stress and receive better healthcare, which could reduce the risk of mental health problems (62). On the other hand, huge treatment expenses caused by mental health problems could lead to poor financial status due to lack of health insurance in rural areas of China (63). We found that those with more frequent major medical conditions were more likely to suffer from insomnia, which is consistent with previous findings (64, 65). Physical and mental distress and economic and psychological burden caused by major medical conditions could all lead to insomnia (47, 66).

We found current drinking was associated with lower risk of depression and insomnia in older adults. In this study, around a third of participants had current drinking behavior, suggesting that drinking is common with social activities (67), particularly in rural areas of China. Better social support through such social activities could reduce the risk of mental health problems (68). In addition, some studies found that the sedative effects of alcohol have a positive effect on certain psychiatric symptoms such as insomnia and anxiety, but the effect is dependent on the amount of alcohol intake (64, 69).

Depression and insomnia were associated with a range of negative consequences such as poor general physical health status and impaired social and cognitive functions; therefore, it is assumed that those with depression, insomnia or comorbid depression and insomnia would have lowered QOL, which is consistent with our findings. Similar findings were also reported in previous studies (70, 71).

The strengths of this study included the relatively large sample size, multi-center design and use of random sampling. However, several limitations should be noted. First, participants were recruited in Anhui province, therefore, the findings could not be generalized to rural areas of other provinces in China. Second, the prevalence of depression and insomnia may be underestimated as older adults with obvious cognitive impairment and brain injury were excluded. Third, the high rate of comorbid depressive and insomnia symptoms may be partly due to the overlap of sleep problems in the CES-D. However, there was only one item on sleep in the CES-D, whereas the ISI measures insomnia symptoms in multiple dimensions (30, 34). Fourth, this study was based on self-report assessment, therefore, the possibility of recall bias could not be excluded. Furthermore, some factors that may be associated with depressive and insomnia symptoms, such as the level of social support or the number of children, were not recorded. Finally, due to the cross-sectional study design, the causality between depression, insomnia and other variables could not be identified.

In conclusion, this study found that depression and insomnia were common in rural older adults. Considering the negative effects of depression and insomnia on QOL and other health outcomes, regular screening and effective treatments should be developed for this population.

## Data Availability Statement

The medical ethics committee of Chaohu Hospital at Anhui Medical University that approved the study prohibits the authors from making the research data set publicly available. Readers and all interested researchers may contact HL (huanzhongliu@ahmu.edu.cn) for details. HL could apply to the medical ethics committee of Chaohu Hospital for the release of the data.

## Ethics Statement

The studies involving human participants were reviewed and approved by medical ethics committee of Chaohu Hospital of Anhui Medical University. The patients/participants provided their written informed consent to participate in this study.

## Author Contributions

HL and Y-TX: study design. J-JY, HC, LX, WN, YZ, SW, and YS: collection, analyses, and interpretation of data. J-JY, HC, and Y-TX: drafting of the manuscript. CN: critical revision of the manuscript. All authors approval of the final version for publication.

## Funding

The study was supported by the University of Macau (MYRG2019-00066-FHS) and the National Clinical Key Specialty Project Foundation (CN).

## Conflict of Interest

The authors declare that the research was conducted in the absence of any commercial or financial relationships that could be construed as a potential conflict of interest.

## Publisher's Note

All claims expressed in this article are solely those of the authors and do not necessarily represent those of their affiliated organizations, or those of the publisher, the editors and the reviewers. Any product that may be evaluated in this article, or claim that may be made by its manufacturer, is not guaranteed or endorsed by the publisher.
